# Role of Adipose Tissue in Inflammatory Bowel Disease

**DOI:** 10.3390/ijms22084226

**Published:** 2021-04-19

**Authors:** Eva Karaskova, Maria Velganova-Veghova, Milos Geryk, Hana Foltenova, Veronika Kucerova, David Karasek

**Affiliations:** 1 Department of Pediatrics, Faculty of Medicine and Dentistry, Palacky University and University Hospital Olomouc, 77900 Olomouc, Czech Republic; Maria.VeghovaVelganova@fnol.cz (M.V.-V.); Milos.Geryk@fnol.cz (M.G.); Hana.Foltenova@fnol.cz (H.F.); 2 Department of Clinical Biochemistry, University Hospital Olomouc, 77900 Olomouc, Czech Republic; Veronika.Kucerova@fnol.cz; 3 Third Department of Internal Medicine—Nephrology, Rheumatology and Endocrinology, Faculty of Medicine and Dentistry, Palacky University and University Hospital Olomouc, 77900 Olomouc, Czech Republic; david.karasek@fnol.cz

**Keywords:** adipokines, visceral obesity, mesenteric fat, inflammatory bowel disease, microbiome

## Abstract

Inflammatory bowel diseases (IBDs), chronic inflammatory disorders affecting the gastrointestinal tract, include Crohn’s disease and ulcerative colitis. There are increasing clinical and experimental data showing that obesity, especially visceral adiposity, plays a substantial role in the pathogenesis of IBD. Obesity seems to be an important risk factor also for IBD disease severity and clinical outcomes. Visceral adipose tissue is an active multifunctional metabolic organ involved in lipid storage and immunological and endocrine activity. Bowel inflammation penetrates the surrounding adipose tissue along the mesentery. Mesenteric fat serves as a barrier to inflammation and controls immune responses to the translocation of gut bacteria. At the same time, mesenteric adipose tissue may be the principal source of cytokines and adipokines responsible for inflammatory processes associated with IBD. This review is particularly focusing on the potential role of adipokines in IBD pathogenesis and their possible use as promising therapeutic targets.

## 1. Introduction

Inflammatory bowel diseases (IBDs), chronic inflammatory disorders affecting the gastrointestinal tract, include Crohn’s disease (CD) and ulcerative colitis (UC). Approximately 25% of IBD patients experience the onset of symptoms before 21 years of age. Pediatric IBD is considered more severe and extensive [1]. The incidence of IBD in both adults and children is increasing worldwide. According to a 2018 review, the highest incidence rates of pediatric IBD were 23 and 15.2 per 100,000 person-years in Europe and North America, respectively [2].

The etiopathogenesis of IBD results from imbalance between genetic predisposition, environmental factors (infections, diet, smoking, drugs, stress and socioeconomic status), and the gut microbiome [3]. A crucial role in bowel inflammation is played by modulation of the cytokine function. Apart from being involved in energy homeostasis, adipose tissue is an endocrine and immune organ significantly contributing to inflammatory processes. A number of adipokines (hormones and cytokines secreted by adipose tissue), especially from the mesenteric fat, can influence the pathogenesis of IBD [4]. Despite progress in this field of research, the etiopathogenetic mechanisms of IBD are not fully elucidated.

## 2. Obesity and IBD

Obesity is characterized by weight gain above normal ranges—body mass index (BMI) ≥ 30 kg/m^2^; overweight is defined as BMI ≥ 25 kg/m^2^. IBD is often associated with underweight (BMI < 18 kg/m^2^); however, numerous studies find overweight and obesity also common in UC/CD patients [5]. Overweight may be present in 20–40% of IBD patients in Western countries, while obesity may affect about 15–40% of this population [6]. Approximately 20% of children with CD and 30% with UC are overweight or obese [7]. There is a direct association between childhood BMI and CD diagnosed before 30 years of age as well as an inverse association between childhood BMI and UC irrespective of age. This supports the hypothesis of obesity being a risk factor for CD and suggests that childhood underweight might be a risk factor for UC. A recently published systematic review of more than 23,000 IBD adult cases also verified obesity as the risk factor of IBD. Obesity and underweight were independently associated with an increased risk of CD, while there was no evident association between BMI and the risk of UC [8].

Patients with IBD have higher abdominal adiposity and less skeletal muscle mass than healthy individuals [9]. Increasing rates of obesity coincide with decreases in lean muscle mass over time [10]. Muscle mass decreases may even result in osteoporosis and more frequent bone fractures in IBD patients [11]. Sarcopenia is common in overweight and obese patients with IBD and was described as a predictor of the need for surgery [12,13]. Sarcopenia also correlated with an increased rate of major postoperative complications, and improved perioperative intervention may diminish this risk [14]. Most overweight patients would not be identified as malnourished using traditional measurement methods. An exact quantification of skeletal muscle mass is possible with abdominal computed tomography [15]. Computed tomography examination of patients after surgical treatment due to refractory IBD observed significant changes in body composition compared to healthy controls. Patients were characterized by increased fat deposition and reduced skeletal muscles. The authors emphasize that patients that are refractory to IBD treatment may have an increased risk of sarcopenic obesity [9].

Obesity, in particular visceral adiposity, might contribute to the course of IBD and its outcome. Adult obese patients with IBD need more frequent hospitalizations compared to those with normal body weight [16]. Similar conclusions were drawn in the pediatric IBD population; both low and high BMI upon diagnosis was associated with a worse course of illness [17]. However, it is not entirely clear whether obese IBD patients are at higher risk of complications, such as surgery, hospitalization, and infection. Studies in patients with Crohn’s disease suggest that overall obesity (as measured by BMI) is not consistently associated with an increased prevalence of IBD-related complications. Similarly, in patients with ulcerative colitis, obesity has not been associated with disease severity [6]. On the contrary, in a study of Malik et al., obese CD patients were approximately 2.5 times more likely to have a poor surgical outcome than patients with CD who were not obese [18]. A body mass index above 25 predicts poorer outcome and a shorter time to first surgery in adult CD patients [19]. Obesity has repeatedly been recognized as a risk factor for perioperative morbidity, which is largely driven by surgical site infections, but it is also by impaired wound healing, potentially increased thromboembolic complications, longer length of hospital stay, and the need for short-term rehabilitation [20]. Obesity has been shown to be a risk factor for conversion from laparoscopic to open surgery [21]. In children, obese pediatric IBD patients may also have a more severe disease course, as indicated by an increased need for surgery [22]. On the contrary, one meta-analysis revealed that obese patients were significantly less likely to undergo IBD-related surgery, receive hormone therapy, and experience hospitalization compared with non-obese patients [23].

Several studies suggest that obesity also negatively impacts overall quality of life in IBD patients [20,21]. For example, recently, a large internet-based study of more than 7000 participants with IBD demonstrated that those who were obese were significantly more likely to have higher rates of anxiety, depression, fatigue, pain, and reduced social function [24]. Whether obesity influences the IBD phenotype is still unclear, few studies exploring this issue have produced conflicting results [20]. One retrospective review suggested that obese patients may have higher rates of perineal disease [25]; other studies found no differences in disease distribution or behavior for CD or UC patients [9,26]. These inconsistencies may be related to significant limitations of BMI as a biomarker of adiposity, as it is unable to differentiate between subcutaneous and visceral adipose tissue [27].

There are data suggesting that obesity does influence the efficacy of specific drugs commonly used to treat IBD. It is associated with the rapid clearance of biologic agents that could result in low trough concentrations and in suboptimal response to biological therapy [5,6]. A reduction in clinical outcome and lower trough levels in response to infliximab [28] and adalimumab [29] have been reported. More recently, trough levels of vedolizumab were found to be reduced by the presence of obesity in patients with IBD [30]. Decreased clinical response and lower 6-thioguanine levels on treatment with azathioprine have been reported, too [21]. On the other hand, in pediatric IBD patients on maintenance infliximab therapy, excess weight gain was not associated with higher weight-based dosing, lower serum trough levels, or increased risk of treatment failure [31]. A pooled data analysis of infliximab-treated IBD patients from landmark clinical trials did not identify any difference in clinical remission or response based on BMI [32]. These findings were supported by a systematic review and meta-analysis demonstrating that obesity was a risk factor for anti-TNF therapy failure in several rheumatic diseases but not in IBD [33].

## 3. Adipose Tissue as an Active Endocrine Organ Visceral Adipose Tissue and IBD

Adipose tissue is an active multifunctional metabolic organ involved in lipid storage and immunological and endocrine activity. It is composed of adipocytes, preadipocytes, macrophages, adipose-derived stromal cells, endothelial cells, fibroblasts, and leukocytes.

**Figure 1:**
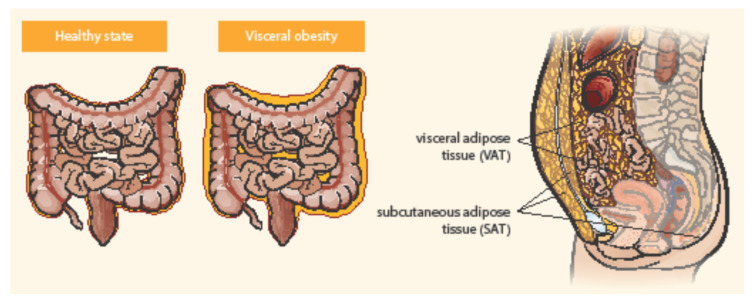
Distribution of abdominal adipose tissue in healthy state and visceral obesity. Adapted from Eder et al. [27].

Adipose tissue is distributed in two main compartments, subcutaneous and visceral—see Figure 1 [27,34]. Exact differentiation of subcutaneous and visceral adipose tissue (VAT) is possible by abdominal magnetic resonance imaging or computed tomography used to measure the visceral fat area and subcutaneous fat area [15,35]. VAT could be a better predictor of disease progression and a risk factor for CD complications than obesity determined by BMI [36]. Computed tomography suggested that the ratio of the area of VAT to that of subcutaneous fat is a good marker for the CD progression and correlated with the postoperative recurrence of CD more strongly than BMI [37]. Pediatric patients with CD were observed with higher VAT volumes than healthy controls and CD-related hospitalization correlated with the increase in VAT volume [38]. Visceral adiposity, as measured by VAT volume, may be associated with a significant increase in the risk of penetrating disease and surgery in CD [39]. VAT is an independent risk factor for the endoscopic recurrence of CD after surgery. Measures of VAT may help to stratify risk in post-operative management strategies [12]. The peri-intestinal compartment of VAT, mesenteric adipose tissue, appears to play a particularly important role in IBD [34].

### 3.1. Mesenteric Adipose Tissue

Mesenteric adipose tissue (MAT) is a biologically very active fat compartment [34,40,41]. MAT may influence the gut barrier function by promoting the innate immune response to the gut flora. MAT may be the principal source of cytokines responsible for inflammatory processes associated with IBDs, for example interleukins (IL): IL-1β, IL-4, IL-6, IL-8, IL-10, tumor necrosis factor-α (TNF-α), adipokines, and other mediators (e.g., angiotensinogen, plasminogen activator inhibitor-1) [40,41]. The degree of cytokine expression has been shown to correlate with adipocyte mass [42]. Furthermore, preadipocytes and adipocytes can express a broad spectrum of functional innate immune receptors, namely Toll-like receptors and nucleotide-binding oligomerization domain-containing proteins 1 and 2 as a reaction to bacterial stimuli [40,43].

MAT is frequently visible by ultrasound imaging, but only magnetic resonance imaging or computed tomography (CT) enterography is an accurate and precise imaging modality for measuring both visceral and subcutaneous adipose tissue. Increased mesenteric fat density evaluated by CT enterography was found to correlate with elevated serum C-reactive protein (CRP) levels in patients with CD [44].

### 3.2. Creeping Fat (Fat Wrapping)

**Figure 2:**
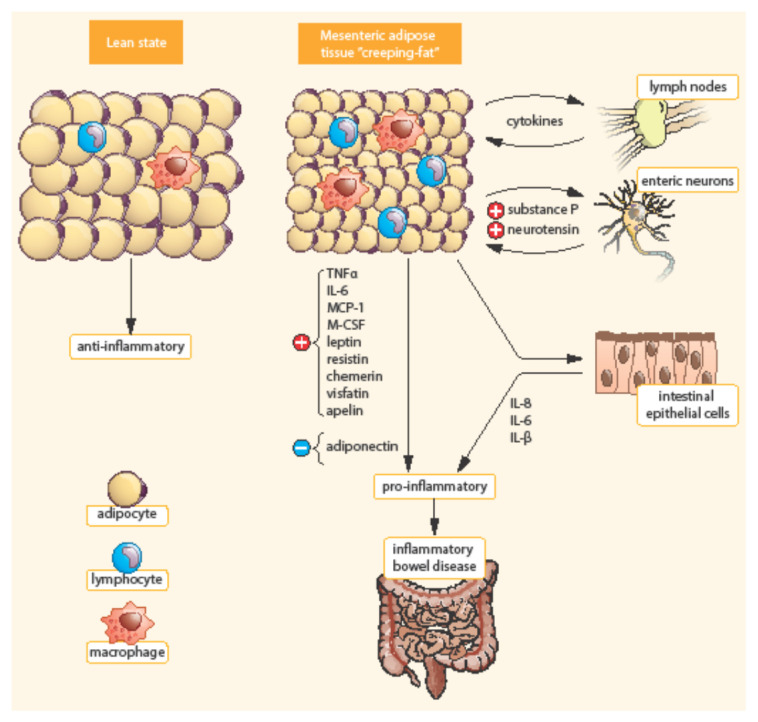
Differences between mesenteric adipose tissue in healthy individuals and patients with IBD. Adapted from [3]. In contrast to lean state, creeping fat is characterized by smaller, highly active adipocytes, with enhanced expression of pro-inflammatory mediators (TNF-α, IL-6, etc.). Mesenteric adipose tissue is infiltrated by immune cells (macrophages, lymphocytes) with enhanced production of chemokines (MCP-1, M-CSF). In addition, pre-adipocytes can differentiate into macrophages. TNF-α, Tumor necrosis factor α; IL, Interleukin; MCP-1, *Monocyte chemotactic protein-1;* M-CSF, Macrophage colony-stimulating factor.

Bowel inflammation penetrates the surrounding adipose tissue along the mesentery in CD patients. MAT extends from the mesenteric attachment toward the root of the mesentery [3]. This hypertrophic mesenteric fat, surrounding the inflamed bowel, is called “creeping fat” or “fat wrapping”—see Figure 2 [34,40]. MAT hypertrophy was described a long time ago, in 1932, by B. B. Crohn as a consistent symptom of CD [45]. Creeping fat is pathognomonic of CD. It consisted of adipocytes, endothelial cells, immune cells, fibroblasts, pre-adipocytes, and stem cells. This activated adipose tissue secretes a broad spectrum of mediators, including cytokines, adipokines, fatty acids, and growth factors. Creeping fat is interpositioned between serosa and muscularis propria, which suggests that adipocytes are in direct contact with intestinal smooth muscle cells. In this way, the presence of creeping fat is associated with muscularis propria hyperplasia, transmural inflammation, and intestinal fibrosis. Fat wrapping, together with muscular hypertrophy and intestinal fibrosis, may participate in the stricturing form of CD [46,47].

In a retrospective study of intestinal resections, fat wrapping was found only in patients with CD and not in resections performed for other diagnoses (intestinal ischemia, tumors, etc.). Fat wrapping was positively correlated with transmural inflammation, fibrosis, stricture formation, and macrophage and lymphocyte perivascular infiltration on histology [48]. The development of mesenteric creeping fat in CD has been hypothesized to be caused by adipocyte hyperplasia rather than hypertrophy [27], resulting in an approximately four-fold increase in the number of mesenteric adipocytes compared with healthy controls [41]. There are also some observations of edematous adipose tissue and enlarged lymph nodes in patients with UC undergoing an abdomino-perineal resection [3]. However, there are probably differences in MAT from patients with ileal CD, colonic CD, and UC [49]. Creeping fat appears to be restricted to ileal specimens; it is less prominent in colonic CD and in UC. Moreover, creeping fat contains in the ileum significantly more fibrotic tissue and T cells than colonic fat from CD or UC patients. Colonic fat from CD patients shared features of both ileal fat from CD patients and colonic fat from UC patients, supporting the concept that these entities should be considered separately [27,49].

A close link and interaction between MAT and the bowel lymphatic system has been described. Lymph nodes are surrounded by perinodal adipose tissue that can also interact with lymph node cells during chronic inflammatory conditions [50]. It is supposed that impaired intestinal or mesenteric lymphatic drainage during intestinal inflammation could favor mesenteric adipocyte hyperplasia and creeping fat [3]. A recent in vitro investigation suggested that activated muscularis propria muscle cells secrete a distinct matrisome, with increased amounts of the extracellular matrix component fibronectin which, through an integrin α5ß1-mediated signaling, induces the migration of preadipocytes out of mesenteric fat and de novo formation of creeping fat [33]. Lymphatic insufficiency was demonstrated to cause adipose tissue accumulation [51]. Mesenteric lymphatic obstruction has been reported in patients with CD undergoing surgery [52].

Recently, a working model for the interactions between lymphatics, fat, and inflammation in CD has been proposed by von der Weid and Rainey [53]. Impaired lymph drainage causes increased fluid volume/pressure in the interstitium and edema. Lymph leak out of dysfunctional lymphatics induces, via lymph factors, preadipocytes to differentiate and form MAT. Conversely, both adipose deposition and interstitial fluid accumulation, alter lymphatic drainage and lead to lymphangiogenesis. Dysfunction of lymphatic vessels also impairs immune cell trafficking to lymph nodes, altering an appropriate immune response. Moreover, mesenteric adipocytes may, by their production of key chemokines in response to inflammatory/bacterial stimuli, arrange the formation of tertiary lymphoid organs rich in both T- and B- memory cells as well as plasma cells [54]. Whole transcriptional analysis also supports the role of CD-associated MAT as a site for T-, B-, and plasma cells activation, which suggests that it could act as a reservoir of memory immune response [55].

### 3.3. Intestinal Microbiota, Visceral Obesity, and IBD

It has been shown that MAT may contribute to the development of CD by reacting to the gut microbiome. Viable bacteria were found in about 20% of mesenteric lymph nodes and adipose tissue in healthy humans, showing the physiological presence of bacteria within adipose tissue. About 95% of the total viable bacteria cultured from mesenteric tissues are physiologically located in adipocytes and only 5% are translocated to mesenteric lymph nodes, indicating that adipocytes might be the main reservoir of bacteria in the mesentery [41]. This suggests that bacteria invading MAT in the course of colitis could affect the local release of inflammatory mediators [3]. The interaction of adipocytes with gut bacteria results in adipocyte hyperplasia, induction of pro-inflammatory genes, and the secretion of chemokines attracting various leukocyte populations. The accumulation of pathogenic bacterial species in mesenteric lymph nodes drives the immune response, resulting in persistent inflammation in the MAT. This aggravates the destruction to the adjacent ileal wall, which further impairs the intestinal barrier and allows more gut bacteria to translocate to the mesentery [27].

Intestinal flora comprises bacteria, viruses, and fungi. It has complicated interactions that probably play a pathogenic role in both obesity and IBD. There are five main phyla of bacteria in the intestinal flora: *Bacteroidetes*, *Firmicutes*, *Proteobacteria*, *Verrucomicrobi*, and *Actinobacteria*. A shift in the microbiome composition leads to dysbiosis in visceral obesity as well as in IBD. There are some discrepancies among studies; however, major differences in bacterial phyla between obesity and IBD seem to be consistent. They comprise the increase in *Firmicutes* and the decrease in *Actinobacteria* in obese subjects compared to IBD cases [56]. In IBD, in addition to the reduction in *Firmicutes*, there is a quantitative and qualitative change in *Bacteroidetes* [57]. Apart from *Actinobacteria*, IBD patients have increased amounts of *Enterobacteriaceae* and *Proteobacteria* [58]. However, there exist not only differences but also similarities in the dysbiosis of obesity and IBD. In both IBD and obesity, there is an increase in *Proteobacteria*, *Ruminococcus gnavus,* and a decrease in *Clostridium* (*leptum*) and *Faecalibacterium prausnitzii* [56].

Compared to UC, VAT in CD is more inflamed and more colonized by intestinal commensal bacteria of type *Enterococcus faecalis*, increasing adipocyte proliferation [59]. In CD, as opposed to UC, the translocation of intestinal bacteria to mesenteric fat depots has been demonstrated, leading to increased CRP secretion in systemically relevant levels in these adipocytes [43]. Significant differences in the microbiota between CD and UC patients were found by using next-generation sequencing in the mesenteric lymph nodes from IBD patients undergoing bowel resection. They with CD were characterized by the overexpression of *Proteobacteria* (containing such pathogens as *E. coli*, *Shigella*, *Salmonella,* and *Helicobacter* spp.). Moreover, the ratio of *Firmicutes*-to-*Bacteroides* was found to be decreased in CD but increased in UC [60].

Although the pathogenic significance of dysbiosis is still not clear, changes in bacterial composition may lead to changes in gut barrier function. Due to the reduction in the number of commensal bacteria, the production of short-chain fatty acids (SCFA) is also reduced. SCFAs are a nutrient for colonocytes, which show a beneficial effect in maintaining the integrity of the intestinal barrier [61]. An important contributor is butyrate, which is a SCFA that is a product of plant polysaccharides fermentation. A low concentration of butyrate enhances the integrity of the intestinal barrier, whereas a high concentration promotes epithelial cell death [62]. The production of another SCFA, acetate, increases protection against the enteropathogen *Escherichia coli 0157:H7* by maintaining epithelial barrier function and restricting the translocation of bacterial toxins to the blood supply [63]. In addition, bacterial lipopolysaccharide (LPS) may trigger low-grade inflammation that contributes to IBD, promotes an inappropriate inflammatory response—leading to an imbalance in local pro- and anti-inflammatory factors, and becomes a factor in promoting IBD [5]. These data show that probably different molecules produced by the bacterial microbiota can modulate the balance between colonic health and disease [62]. Bacteria involved in the production of SCFA such as *Faecalibacterium prausnitzii*, and *Bifidobacteria* are reduced in IBD, but reporting of such changes is somewhat variable [56].

Recently, Serena et al. tested the hypothesis that creeping fat may be a bacterial reservoir in patients with CD. They found a microbiome signature within creeping fat and MAT from CD patients, but not in subcutaneous fat. *Proteobacteria* was the most abundant phylum in both creeping fat and MAT, and it was positively correlated with fecal calprotectin and CRP. Notably, the clinical status of patients seemed to be related to the microbiome signature, as those with the inactive disease showed a reduction in the abundance of pathogenic bacteria. These findings demonstrated that microbiota dysbiosis associated with CD pathophysiology is reflected in VAT, which might contribute to disease by potential bacterial translocation across a disrupted intestinal barrier [64].

### 3.4. Mesenteric Fat as a Source of Inflammatory Peptides

Adipocytes are an extrahepatic source of CRP. In a study by Anty et al., CRP gene expression was not only increased in the liver but also in the adipose tissue of obese patients compared with controls subjects. In human adipose tissue, levels of CRP mRNA were positively correlated with those of IL-6, and the CRP expression was enhanced in vitro by IL-6 and lipopolysaccharides [65]. Mesenteric fat is an important source of CRP also in CD patients. In a study by Peyrin-Biroulet et al., CRP expression was higher in the mesenteric fat of CD patients than those with UC; the samples were obtained during surgery. Increased mesenteric fat density correlated with serum CRP levels in CD [43]. CRP production by mesenteric adipocytes may be triggered by inflammatory and bacterial stimuli during bacterial translocation to mesenteric fat. It has been hypothesized that increased cytokine production in the inflamed mesentery, together with translocating bacteria, may trigger CRP production by mesenteric adipocytes in CD patients.

Moreover, the VAT expresses the whole machinery of inflammatory peptides, including classical cytokines (IL-1β, IL-6, TNF-α), chemokines (monocyte chemoattractant protein-1, C-C motif chemokine ligand 2), complement components (C1q, C3a), Toll-like receptors (TLRs), nucleotide-binding oligomerization domain (NOD)-like receptors (NLRs), and C1q/TNF-related proteins [66]. Thus, VAT could link innate immune reactions during gut inflammation to adjacent adipose tissue alterations such as creeping fat [67]. Intestinal adipocytes residing within the VAT adjacent to inflamed gut express major functional components of the innate immune recognition system, such as TLRs and NODs/NLRs. Consequently, visceral adipocytes are able to sense a wide variety of microbial components that cross the disturbed intestinal barrier seen in IBD [68]. Thus, the observed inflammatory transformation of VAT seems to be a consequence rather than the cause of IBD. The physiological meaning behind this mechanism is most likely to provide an additional antimicrobial barrier surrounding the affected gut. This adipose tissue barrier might reduce the risk of intestinal perforation, bacterial translocation to the peritoneum, and finally, systemic inflammation and sepsis [69].

VAT also actively participates in immune responses via secretion of fat-derived hormones, so-called adipokines.

## 4. Adipokines

Adipokines represent a group of mediators primarily released by adipocytes that modulate a variety of metabolic functions in the adipose tissue, liver, brain, muscles, pancreas, and immune system [70]. Adipokines play a central role in controlling energy metabolism. Regulatory immune function has been identified in many of them. The role of several adipokines in the creeping fat as well as in intestinal inflammation was recently explored [71].

### 4.1. Adiponectin

Adiponectin is a hormone exclusively secreted by adipocytes at a level inversely proportional to fat mass [41]. Adiponectin acts as an anti-diabetic and anti-atherogenic factor [72]. It is a key mediator in the pathogenesis of chronic inflammation-related metabolic diseases such as atherosclerosis or type-1 diabetes. Adiponectin activates adiponectin receptor-1 and adiponectin receptor-2. The expression of adiponectin receptors has been reported on human monocytes, B-lymphocytes, and NK cells. Adiponectin induces the direct inhibition of pro-inflammatory pathways, including those regulated by TLRs, through inhibition of nuclear factor-kappa B (NF-κB) in several cell types. Pro-inflammatory cytokines (e.g., TNF-α and IL-6) suppress adiponectin secretion in adipocytes. Adiponectin also increases the secretion of anti-inflammatory cytokines such as IL-10 and IL-1 receptor antagonist by human monocytes, macrophages, and dendritic cells. Therefore, adiponectin acts as an anti-inflammatory mediator [3]. Hypoadiponectinemia is associated with obesity, diabetes, hypertension, mixed dyslipidemia, metabolic syndrome, nonalcoholic steatohepatitis, coronary artery disease, and others [73].

Adiponectin exerts anti-inflammatory effects through the modulation of signaling pathways and may therefore play a critical role in IBD severity and treatment [4]. However, human studies have yielded conflicting results. In one study, mesenteric adiponectin expression was lower in patients resected for CD than those resected for other reasons (with the normal distal ileum). The authors suppose that the lower levels of serum and mesenteric adiponectin in active CD suggest a defective regulation of anti-inflammatory pathways in CD pathogenesis [74]. By contrast, another study found significantly increased tissue concentrations and release of adiponectin (adiponectin mRNA levels) in hypertrophied MAT of CD patients as compared with normal MAT of CD patients, UC patients, and controls [75]. Serum adiponectin levels were also found decreased [74,76,77], increased [78,79], or unchanged [80,81,82] in IBD patients. Circulating adiponectin levels in patients with IBD treated with anti-inflammatory therapy do not seem to be affected [80,83]. The discrepancy in these results in part could be explained by small data cohorts used in some of the studies, inadequate controls, and different treatment status of the patients [62].

The results of many studies suggest that adiponectin can promote the anti-inflammatory response through the modulation of phagocytosis or suppression of pro-inflammatory cytokines, such as TNF-α and IL-6, by suppressing NF-κB signaling [84]. However, some studies have revealed that the role of adiponectin in intestinal inflammatory disease may be pro-proliferative and pro-inflammatory through the activation of some kinases and NF-κB signaling in colonic epithelial cells [85]. These controversial findings support the concept that adiponectin plays an important role in maintaining intestinal homeostasis, but its exact action in bowel inflammation remains unclear and more studies need to be elucidated.

### 4.2. Leptin

Leptin (also known as OB protein) was initially identified as a hormone and satiety factor. Its name is derived from the Greek word *leptos,* which means “thin” [86]. This peptide, encoded by the ob gene, is primarily secreted by adipocytes and is a critical hormone that controls body weight due to its central effects [87]. Leptin mediates several physiological functions including elevated blood pressure, tumorigenesis, cardiovascular pathologies, and enhanced immune response in many autoimmune diseases [88].

This adipokine is overexpressed in the MAT of CD and UC subjects. All studies investigating the leptin secretion/mRNA expression in MAT in IBD patients found unequivocally increased levels [67]. Leptin mRNA levels were shown to be significantly higher in the MAT of CD and UC patients than in controls [88]. However, circulating serum leptin levels were found decreased [78,80,81,89], increased [77,90,91], or unchanged [74,76,82,92,93] in IBD patients. One study documented low serum leptin associated with an increased risk of IBD and also with an increased disease activity on endoscopy [94]. These different findings reflect different treatment status of the IBD patients, small cohorts of included participants, and different disease subgroups (CD versus UC). Moreover, the data from treatment-naïve individuals is not always available, and the control groups are not homogenous as they may include healthy individuals, IBD patients in remission, or patients with gastrointestinal diseases other than IBD [27,62,67].

Leptin exerts strong pro-inflammatory effects by synergizing with TNF-α to activate macrophages and generate reactive oxygen species in neutrophils [3,27]. It stimulates and promotes the proliferation of human peripheral blood mononuclear cells. It is necessary for appropriate T-cell response, increases the proliferation of CD4+ T cells, and also promotes dendritic cell differentiation. Inflamed colonic epithelial cells express and release leptin into the intestinal lumen. In response to luminal leptin, model intestinal epithelia critically activate the NF-κB, which is a key signaling system to pro-inflammatory stimuli; luminal leptin can induce colonic epithelial neutrophil infiltration [86]. Recently, Ziegler et al. have described a patient with the unique combination of acquired generalized lipodystrophy and Crohn’s disease featuring a lack of adipose tissue, leptin deficiency, and intestinal inflammation. Recombinant leptin exerts diverse pro-inflammatory effects on immune cell differentiation and function, including the metabolic reprogramming of immune cells and the induction of TNFα, ultimately aggravating Crohn’s disease, which can be reversed by anti-TNFα therapy. These results indicate that leptin is required for human immune homeostasis and contributes to autoimmunity in a TNFα-dependent manner [95].

### 4.3. Resistin

Resistin is an adipokine expressed and produced in adipocytes and immune cells, mainly peripheral blood mononuclear cells and macrophages [4]. Resistin is named after its resistance to insulin. This adipokine plays a role in the murine pathogenesis of obesity and diabetes. Circulating resistin levels are decreased by the anti-diabetic drug rosiglitazone and increase in diet-induced and genetic forms of obesity. Insulin-stimulated glucose uptake by adipocytes is enhanced by the neutralization of resistin and is reduced by resistin treatment [96]. In humans, resistin may be a link between inflammation and insulin resistance [3].

This adipokine has been associated with inflammatory processes because its expression in adipose tissue is induced by pro-inflammatory cytokines (IL-1β, IL-6, and TNF-α) via the NF-κB signaling pathway [21,27]. Resistin acts as a pro-inflammatory factor. Several studies have demonstrated circulating levels of resistin increased in patients with IBD [80,82]. Resistin levels was an independent predictor of disease activity in patients with CD according to one study [97]. Another study did not find an association between resistin and endoscopic activity of IBD [94]. Anti-inflammatory therapy in patients with IBD significantly reduced serum levels of resistin [80,83]. However, there was no apparent differences in serum resistin between IBD and other diseases characterized by chronic inflammation, which may indicate it is a non-specific marker of inflammation [27].

### 4.4. Chemerin

Chemerin is produced by adipocytes and displays dual functions in immune response and metabolism control. Chemerin expression and secretion increase dramatically with adipogenesis. It is a pro-inflammatory cytokine implicated in adipocyte differentiation and metabolism, insulin resistance, and blood pressure control [98]. Chemerin has been shown to promote the chemotaxis of dendritic cells and macrophages. Therefore, it serves as a chemoattractant for cells of innate immunity [71]. Chemerin levels correlate with serum concentrations of TNF-α, IL-6, and CRP, and they may reflect the inflammatory status associated with obesity [3]. Chemerin mRNA expression in tissue biopsies from patients with UC correlated with disease activity [99]. Moreover, circulating levels of chemerin was increased in patients with IBD compared with healthy controls [79]. This finding was confirmed by some [100] but not all studies [80].

### 4.5. Visfatin

The term visfatin was adopted because of its predominant production in VAT. Although initially reported to be produced preferentially by VAT [101], now, it is known to be secreted also by other cells [102]. Visfatin expression is strongly correlated with the amount of visceral fat and MAT. It could be associated with the pathogenesis of IBD by inducing inflammatory pathways. Visfatin stimulates human leukocytes to produce pro-inflammatory cytokines including TNF-α, IL-6, and IL-1β [4]. Moreover, it was found to act as a potent chemotactic factor for monocytes and B cells and an activator of antigen-presenting cells, phagocytes, and T cells [27]. Circulating visfatin levels have been found to be significantly elevated in both CD and UC patients compared with controls. Visfatin was also higher in UC patients in the activation period than in post-treatment remission patients and healthy controls [103]. Its higher expression was found in colonic biopsies from children with IBD naïve to therapy [104]. Visfatin mRNA expression was significantly upregulated in the colonic tissue of patients with CD or UC [105]. Multiple actions in inflammation and cell proliferation make visfatin a potential therapeutic target [27].

### 4.6. Apelin

Apelin is a relatively novel adipokine produced and secreted by adipocytes [106]. Apelin mRNA levels in adipocytes and its plasma concentrations were increased in various mouse models of obesity. Apelin is also related to the pathophysiology of oxidative stress and inflammation [107]. Its production is upregulated in colonic tissues of IBD patients. Synthetic apelin significantly enhanced the proliferation of mice colonic epithelial cells, suggesting that its enhanced expression in the intestinal recovery stage may result in the repair of the intestinal epithelium in rodent colitis models and in patients with IBD [108]. Apelin is highly produced in the adipose tissue of patients with CD compared with that from controls, and the systemic delivery of apelin significantly ameliorates the disease activity by downregulating inflammatory cytokines such as TNF-α and IL-6 [109]. Further studies have revealed the critical role of apelin signaling in the polarization of endothelial cells [110], in lymphatic endothelial cell migration [111], and in inflammation inhibition by enhancing lymphatic function [112]. These results demonstrated that apelin may have a supportive role in intestinal lymphatic drainage of IBD.

### 4.7. Other Adipokines and Hormones Related to IBD

Ghrelin, a gut–brain peptide, directly and indirectly participates in biological activities including energy metabolism, control of food intake, and stimulation of growth hormone release [4]. Ghrelin plays a role in modulating immune responses and inflammatory processes. It antagonizes leptin by inhibiting leptin-induced pro-inflammatory responses in macrophages and T cells, reducing the expression of pro-inflammatory cytokines (including TNF-α, IL-1β, IL-6, and IL-8), and decreasing the expression of leptin in the gastrointestinal tract [113]. Ghrelin levels were shown to be higher in active IBD cases [76,83,114,115,116]. No significant differences of serum ghrelin levels were found in CD patients in remission compared to healthy controls [92]. Another study did not observe ghrelin levels to be associated with a risk of IBD nor with a disease activity on endoscopy [94]. Therefore, similar to other adipokines, systemic ghrelin levels do not change consistently in IBD. Moreover, there is a possible link between ghrelin levels and the loss of appetite from which patients with IBD suffer [116].

Omentin-1 is expressed predominantly in omental tissue and plays an anti-inflammatory role by inhibiting TNF-α in vascular endothelial cells [117]. It was found to be decreased in obesity, type 2 diabetes, coronary artery disease [27,117], and also in IBD [118]. Ometin-1 levels correlated inversely with disease activity. Moreover, omentin-1 mRNA expression was found to be reduced in colonic tissue from active CD patients [118].

Serum concentration of vaspin is associated with obesity and insulin resistance in humans [102]. No difference in vaspin levels between IBD patients and healthy controls was observed [100]. There are reports that serum levels of retinol-binding protein 4, another adipokine linked to the metabolic syndrome [73,119], has been reported to be elevated in IBD patients [76] and inversely correlated with disease activity [120]. Finally, meteorin-like, a novel adipokine with insulin sensitizing and anti-inflammatory properties, has been recently detected as decreased in patients with IBD [121]. Meteorin-like levels were inversely related with BMI, TNF-α, and IL-6, which suggested a possible relation with the inflammatory process in IBD.

## 5. Adipokines and IBD Treatment

### 5.1. Adipokines for Therapeutic Use

Adiponectin is intended as a potential therapeutic modality, because it plays an important role in maintaining intestinal homeostasis [36]. A plant-derived homolog of adiponectin exerted protective effects during murine dextran sodium sulfate-induced (DSS) colitis [122]. In addition, the administration of adiponectin by adenovirus infection significantly reduces DSS colitis severity in mice [123]. Adiponectin attenuated the stress signals and apoptotic status in colonic epithelial cells and reducing adiponectin resistance or using adiponectin homologs may become therapeutic options in IBD [124]. However, the use of adiponectin as a pharmacological agent is difficult due to its multiple forms and high levels in the blood [125].

The emerging role of a leptin antagonist as a potential therapeutic option for IBD has been also described [87]. Leptin antagonists may ameliorate chronic colitis in IL-10 deficient mice [126]. In addition to adiponectin, apelin significantly promotes the proliferation of epithelial cells in intestinal tissues [108] and also plays a critical role in stabilizing the development of lymphatic vessels [111,112]. The administration of apelin can enhance the lymphatic function of intestinal tissues and reduce colitis in IL-10-deficient mice model [127]. Recently, FK886, a small molecule inhibitor of intracellular visfatin, showed an improved course of DSS colitis in mice with a shift in macrophage sub-populations toward an anti-inflammatory M2 phenotype [128]. These results denote that adipokines may serve as future therapeutic targets for patients with IBD [36].

### 5.2. Adipokines and Anti-TNF Therapy

TNF-α is a critical mediator of inflammatory process in IBD pathogenesis. Associations between TNF-α, VAT, and adipokines have been described. A study by Karmiris et al. showed no significant alterations in serum levels of leptin and adiponectin, whereas serum resistin levels were significantly decreased after infliximab therapy in IBD patients, suggesting its possible pro-inflammatory status in IBD and role as a marker of successful therapy [83]. In patients with CD, leptinemia significantly increased at one and four weeks after infliximab administration, suggesting that TNF-α exerts major inhibitory actions on leptin production in these patients [129]. In the pediatric population, an association between anti-TNF therapy and changes in circulating adipokines has been recently reported. During infliximab induction treatment, transient elevation of adiponectin has been described, with a subsequent decline. The study authors concluded that the marked early increase of the potent anti-inflammatory adiponectin may contribute to the rapid response to infliximab in CD patients [130]. Conventional therapy (prednisone, 5-aminosalicylate, and azathioprine for CD patients) in IBD patients was associated with a decrease in the serum visfatin in CD subjects and a decrease in resistin levels in UC patients. There were no differences in concentrations of other adipokines—leptin, adiponectin, and chemerin [80].

## 6. Conclusions

There are increasing clinical and experimental data showing that the adipose tissue, especially VAT, is involved in the pathogenesis of IBD. VAT exhibits pro-inflammatory, immunoregulatory, and endocrine activity. Obesity seems to be an important risk factor also for IBD disease severity and clinical outcomes. The exact mechanisms by which obesity mediates these effects are not exactly clear. However, this is probably achieved by VAT participating in immune responses to gastrointestinal microbiota and by secreting a number of key mediators with inflammation-modulating activities leading to changes in local cytokine and hormone production. Adipokines participate in many functions, including the regulation of lipid metabolism, insulin sensitivity, inflammation, angiogenesis, hemostasis, and immunity. There are increasing clinical and experimental data showing that the adipokines may act as important mediators involved in IBD. Further research is still required to fully understand the potential role of adipokines in IBDs pathogenesis and to clarify their possible use as promising therapeutic targets.

## Data Availability

The data presented in this study are available on request from the corresponding author.
